# ﻿Caddisflies (Trichoptera) checklist and a new species of *Helicopsyche* von Siebold, 1856, from the Brejo de Altitude de Triunfo, a relict rainforest within the Caatinga domain, Northeast Brazil

**DOI:** 10.3897/zookeys.1111.77541

**Published:** 2022-07-11

**Authors:** Amanda Cavalcante-Silva, Rafael Pereira, Adolfo Ricardo Calor

**Affiliations:** 1 Laboratório de Entomologia Aquática, PPG Biodiversidade e Evolução, Instituto de Biologia, Universidade Federal da Bahia, Rua Barão de Jeremoabo, 147, Campus Ondina, CEP 40170-115, Salvador, Bahia, Brazil Universidade Federal da Bahia Salvador Brazil

**Keywords:** Aquatic insects, distribution, Helicopsyche (Feropsyche), larvae, semaphoronts, snail-case caddisfly, taxonomy

## Abstract

Brejos de Altitude are evergreen seasonal forests, associated with plateau regions in the middle of the Caatinga domain in Northeast Brazil, which possibly acted as biological corridors between the Atlantic Forest and the Amazon rainforest during the Pleistocene. The first entomological survey in the highest point in the state of Pernambuco, Brazil, the Brejo de Altitude de Triunfo, was implemented and resulted in a checklist of caddisflies with six families, nine genera, and eleven species, including a new species. *Helicopsycheralphi***sp. nov.** is described and illustrated, based on all semaphoronts. A key to Brazilian Helicopsyche (Feropsyche) Johanson, 1998 species is also provided. In addition to the caddisfly survey in the Brejos de Altitude, the results include new records for the state, region, and also for the country. Thus, this study updates the number of species in the Brazilian Northeast region and Pernambuco state to 169 species and 43 species, respectively.

## ﻿Introduction

The Caatinga domain is a mosaic of xerophyte forest of 912.529 km^2^ in Northeastern Brazil (da Silva et al. 2017), delimited by the Atlantic Forest, Amazon rainforest and Cerrado domains. Previously, the area that today comprises Caatinga was a connection between the Atlantic Forest and Amazon rainforest (Santos et al. 2007; Batalha-Filho et al. 2013). The palynological profile from the late Pleistocene (0.9 Mya) in the Caatinga domain revealed a high concentration of pollen of taxa found in the present Atlantic Forest and Amazon rainforest, probably reflecting a connection of these domains during this period (Costa et al. 2017; Ledo and Colli 2017). The initial separation of these regions possibly occurred because of the Andean uplift, which changed the climate and consequently the vegetation of South America (Morley 2000). These changes led to the modification and emergence of a “dry diagonal”, an area with more xeric habitats, separating the two forests (Costa et al. 2017). This splitting process started in the Miocene (5.6–23 Mya), but the total separation occurred only in the early Pleistocene (the last 5.5 Mya) (Batalha-Filho et al. 2013; Costa et al. 2017; Ledo and Colli 2017). Subsequently, the Caatinga domain has been characterized by a xerophyte forest mosaic, with some islands of humid tropical forests, named Brejos de Altitude (Andrade-Lima 1982; Ledo and Colli 2017). Possibly due to this recent separation many sister species and lineages have disjunct distributions in the Atlantic Forest and Amazon rainforest (Borges-Nojosa and Caramaschi 2003; Batalha-Filho et al. 2013; Ledo and Colli 2017; Silveira et al. 2019).

Brejos de Altitude represent forest refuges enclaved in the Caatinga domain (Pereira-Filho and Montingelli 2011). These areas are a mosaic composed of Atlantic Forest and Amazon biotic components, and they have climatic, edaphic, and topographical features different from their semiarid surroundings (Borges-Nojosa and Caramaschi 2003), and harbor a peculiar biodiversity of amphibians and reptiles (e.g., Pereira-Filho and Montingelli 2011; Castro et al. 2019a, b; Quirino et al. 2019), insects (e.g., Silva et al. 2007; Santos et al. 2011; Silva et al. 2019), and plants (e.g., Rodal et al. 2005; Machado et al. 2012; Araujo et al. 2019). The Brejos de Altitude possibly originated from climatic fluctuations that occurred during the Pleistocene, allowing the expansion of Atlantic Forest into currently semiarid locations in areas with a favorable microclimate during the shrinkage process (Behling et al. 2000; Auler et al. 2004; Silveira et al. 2019).

Brejos de Altitude environments also play an important role in freshwater flow, and as a consequence of orographic rains, several headwater streams emerge from them (Andrade-Lima 1982; Araújo et al. 2007). Headwater streams represent essential habitats for taxa primarily associated with these environments, such as some families of Trichoptera (Richardson 2019).

Trichoptera is the most diverse order of strictly aquatic insects, with ~ 16,300 extant species, 632 genera and 63 families (Morse 2022). Of these, ~ 3,300 species, 25 families, and 155 genera were recorded in the Neotropical region (Holzenthal and Calor 2017; Morse et al. 2019). In Brazil, ~ 900 species of Trichoptera are recorded (Santos et al. 2022). Although our knowledge of caddisflies from Brazil has increased in the last years (Vilarino and Calor 2017), the Atlantic Forest and Amazon rainforest contain the most concentrated species records, possibly as a consequence of research groups established in these regions for a longer time. On the other hand, our knowledge of caddisflies from the Caatinga has increased, with 77 species (14 endemic) now known (Santos et al. 2022). Currently there are 39 species of Trichoptera reported for Pernambuco state (Souza et al. 2013a; Gomes and Calor 2019; Pereira et al. 2020), of which three are representatives of Helicopsychidae: Helicopsyche (Cochliopsyche) clara (Ulmer, 1905), Helicopsyche (Feropsyche) tapadas Denning, 1966, and Helicopsyche (Feropsyche) vergelana Ross, 1956 (Souza et al. 2013a; Pereira et al. 2020). In all Brejos de Altitude, only three caddisfly species [*Macrostemumhyalinum* (Pictet, 1836), *Oxyethiratica* Holzenthal & Harris, 1992, and *Atopsycheantisuya* Schmid, 1989] have been recorded, in França et al. (2013), Souza and Santos (2017), and Gomes and Calor (2019), respectively.

Trichoptera are holometabolic insects, presenting an egg, larva (generally five instars), pupa and adult stage (Holzenthal et al. 2015). For most insects, the morphology of females and immatures is little known, because descriptions and identification tools have a male bias (Yeo et al. 2018). This shortfall of knowledge of the semaphoronts (Haeckelian shortfall) (Faria et al. 2020) is exaggerated in *Helicopsyche*, where only 19 immature stages and 63 adult females of the 130 valid species from the Neotropical region are known (four immatures and 13 adult females from Brazil), but most have no description of these semaphoronts (only 17 immatures and 25 adult females have descriptions) (Johanson 2002; Holzenthal and Calor 2017; Morse 2022). The subgenus H. (Feropsyche) has knowledge shortfalls of the species (Linnean shortfalls), since ~ 40% were described only in the 21^st^ century (e.g., Johanson 2003; Johanson and Malm 2006; Johanson and Holzenthal 2010; Rueda-Martín and Miranda 2015; Vilarino and Calor 2017; Dumas and Nessimian 2019), and many species to be described. Considering the significant reduction of vegetation in the Brazilian Northeast Atlantic Forest, the Brejos de Altitude constitute areas of high priority for conservation (SOS Mata Atlântica & INPE 2019; Pereira-Filho et al. 2020). Here we present the first study of caddisfly fauna from a Brejo de Altitude with a commented checklist. Additionally, we present the description of *Helicopsycheralphi* sp. nov., based on adult males and females and immature stages, and a key to Brazilian Helicopsyche (Feropsyche) species.

## ﻿Materials and methods

**Figure 1.**
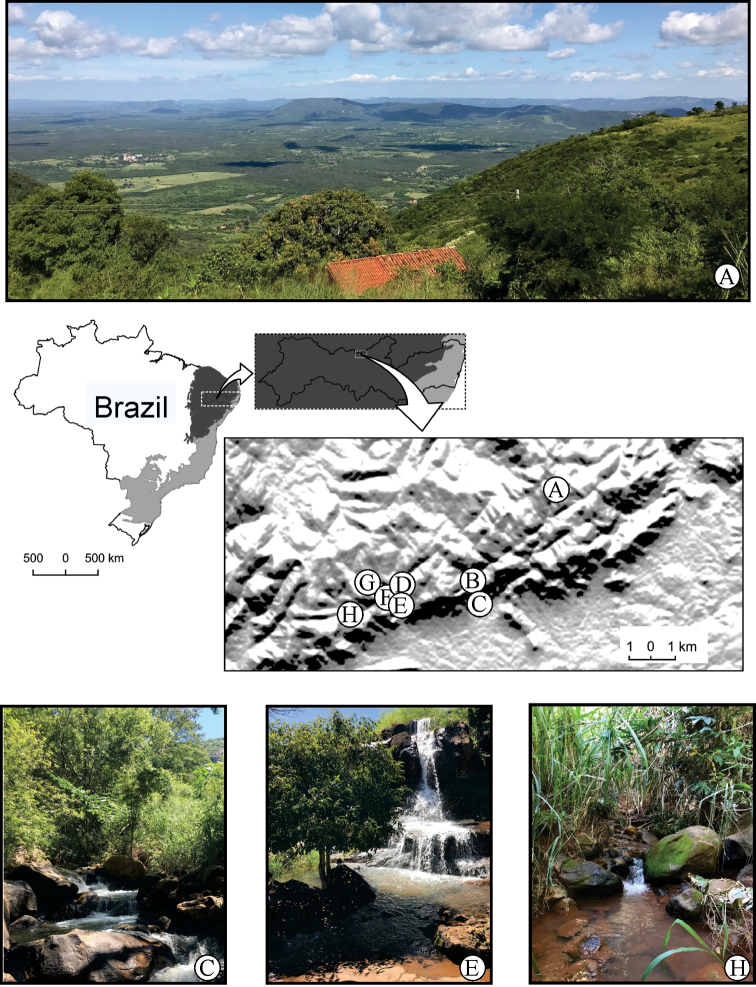
Distribution map of the sampling sites in the Brejo de Altitude de Triunfo, Pernambuco state, Brazil **A** Pico do Papagaio stream **B** Grito stream **C** Laje stream **D, E, F** Pinga stream **G** Alfinim stream **H** Icó stream.

The Brejo de Altitude de Triunfo (07°50'17"S, 38°06'06"W) is located in the Baixo Pajeú region and represents the highest altitude mountain in the Pernambuco state, Brazil, with altitude of 500–1,260 m. The area is predominantly composed of seasonal semideciduous forest, exhibiting average rainfall of 1,222 mm/year, with higher rainfall occurring in March and April (Tabarelli and Santos 2004). Specimens were collected between 2017 to 2019 from the following sites: (A) Pico do Papagaio stream; (B) Grito stream; (C) Laje stream; (D, E, and F) Pinga stream; (G) Alfinim stream, and (H) Icó stream (Fig. 1, Table 1). Adults were collected by means of light bulbs (ultraviolet and white lights) attached to a white sheet, UV light pan trap (Calor and Mariano 2017), and Malaise trap. Immature stages were collected manually. All specimens were preserved in 80% ethanol. For each collector, an acronym was designated, as follows: ACS for Amanda Cavalcante-Silva and RP for Rafael Pereira.

**Table 1.** Collection data from Brejo de Altitude de Triunfo, with the sample of each collection site, geographic coordinates, elevation, date, traps (LPT = UV Light Pan Trap, MAL = Malaise and WSA = White Sheet Attraction), and acronyms of collectors.

Sample	Collection sites	Geographic coordinates	Elevation (a.s.l.)	Date	Trap	Acronyms of collectors
A1	Pico do Papagaio stream	7°49'36"S, 38°3'32"W	1050 m	02.v.2019	LPT	ACS, RP
B1	Grito stream	7°51'41"S, 38°5'25"W	740 m	07.viii.2018	LPT	ACS
B2		7°51'41"S, 38°5'25"W	740 m	09.viii.2018	LPT	ACS
B3		7°51'41"S, 38°5'25"W	740 m	09.x.2018	LPT	ACS
C1	Laje stream	7°52'13"S, 38°5'18"W	580 m	07.viii.2018	LPT	ACS
C2		7°52'13"S, 38°5'18"W	580 m	08.ix.2018	LPT	ACS
C3		7°52'13"S, 38°5'18"W	580 m	10.x.2018	LPT	ACS
C4		7°52'13"S, 38°5'18"W	580 m	i.2019	MAL	ACS
C5		7°52'28,2"S, 38°8'15,6"W	570 m	02.v.2019	LPT	ACS, RP
C6		7°52'28,5"S, 38°8'13,6"W	560 m	02.v.2019	LPT	ACS, RP
C7		7°52'28,5"S, 38°8'15,3"W	860 m	03.v.2019	LPT	ACS, RP
D1	Pinga stream	7°52'3"S, 38°7'13"W	890 m	16.xii.2017	LPT	ACS
D2		7°52'3"S, 38°7'13"W	890 m	18.xii.2017	LPT	ACS
D3		7°52'3"S, 38°7'13"W	890 m	21.ix.2017	LPT	ACS
D4		7°52'3"S, 38°7'13"W	890 m	07.ii.2018	LPT	ACS
D5		7°52'3"S, 38°7'13"W	890 m	09.ii.2018	LPT	ACS
D6		7°52'3"S, 38°7'13"W	890 m	06.viii.2018	WSA	ACS
D7		7°52'3"S, 38°7'13"W	890 m	06.viii.2018	LPT	ACS
D8		7°52'3"S, 38°7'13"W	890 m	07.viii.2018	LPT	ACS
D9		7°52'3"S, 38°7'13"W	890 m	21.viii.2018	LPT	ACS
D10		7°52'3"S, 38°7'13"W	890 m	ix.2018	MAL	ACS
D11		7°52'3"S, 38°7'13"W	890 m	09.x.2018	LPT	ACS
D12		7°52'3"S, 38°7'13"W	890 m	03.ii.2019	LPT	ACS
D13		7°52'3"S, 38°7'13"W	890 m	07.ii.2019	LPT	ACS
D14		7°52'3"S, 38°7'13"W	890 m	09.ii.2019	LPT	ACS
D15		7°52'3"S, 38°7'13"W	890 m	10.ii.2019	LPT	ACS
D16		7°52'3"S, 38°7'13"W	890 m	11.iii.2019	LPT	ACS
D17		7°52'3"S, 38°7'13"W	890 m	16.iv.2019	LPT	ACS
D18		7°52'5,5"S, 38°7'15,6"W	870 m	01.v.2019	LPT	ACS, RP
D19		7°52'4,7"S, 38°7'15,3"W	860 m	01.v.2019	LPT	ACS, RP
D20		7°52'5,5"S, 38°7'15,7"W	865 m	01.v.2019	LPT	ACS, RP
D21		7°52'3,2"S, 38°7'13,8"W	840 m	01.v.2019	LPT	ACS, RP
D22		7°52'3,2"S, 38°7'13,8"W	840 m	02.v.2019	LPT	ACS, RP
E1	Alfinim stream	7°51'44"S, 38°7'52"W	940 m	08.viii.2018	LPT	ACS
E2		7°51'44"S, 38°7'52"W	940 m	08.viii.2018	WSA	ACS
F1	Icó stream	7°52'28,8"S 38°8'15,8"W	800 m	01.v.2019	LPT	ACS, RP
F2		7°52'28,5"S, 38°8'15,3"W	810 m	02.v.2019	LPT	ACS, RP
F3		7°52'28,5"S, 38°8'15,8"W	800 m	02.v.2019	LPT	ACS, RP

The map with collection sites was created using QGIS 3.4.15 and finalized in Corel Draw X5. The species distribution data were obtained from Holzenthal and Calor (2017) for the Neotropical region and Queiroz et al. (2020) and Santos et al. (2022) for Brazil. New records for Pernambuco state are indicated in the species distribution. Genitalia of males and females were diaphanized in 10% KOH solution or lactic acid (Betten 1934; Blahnik and Holzenthal 2004; Blahnik et al. 2007) and stored in microtubes with glycerin. Association between immature and adult stages was done using the metamorphotype method (Milne 1938).

The illustrations were made with the aid of a microscope equipped with a camera lucida, scanned, and finalized in Adobe Illustrator CS6. Microphotographs were made with a Leica stereoscope equipped with a digital camera, Nikon model DS-Fi1 and finalized in Corel Draw X5. Descriptions were made using the DELTA system (Dallwitz et al. 1999). The terminology applied to the morphological structures of adults follows Johanson (1998), with adaptations of Holzenthal et al. (2016), and immature follows Monson et al. (1988) and Waringer et al. (2017). The type specimens will be deposited at the following institutions: Museu de Zoologia da Universidade São Paulo, São Paulo (**MZSP**), Museu de História Natural da Bahia, Universidade Federal da Bahia, Salvador (**UFBA**), Coleção Entomológica Prof. José Alfredo Pinheiro Dutra, Departamento de Zoologia, Universidade Federal do Rio de Janeiro, Rio de Janeiro (**DZRJ**), and Instituto Nacional de Pesquisas da Amazônia, Manaus (**INPA**), as indicated in the material examined section.

## ﻿Results

### ﻿ Helicopsyche (Feropsyche) ralphi sp. nov.

Taxon classificationAnimaliaTrichopteraHelicopsychidae

D0B9A23F-5104-5CDE-A8EE-3DE5A93BF24E

http://zoobank.org/15602B59-C063-4007-82C0-9141BD06D2BE

**Figure 2.**
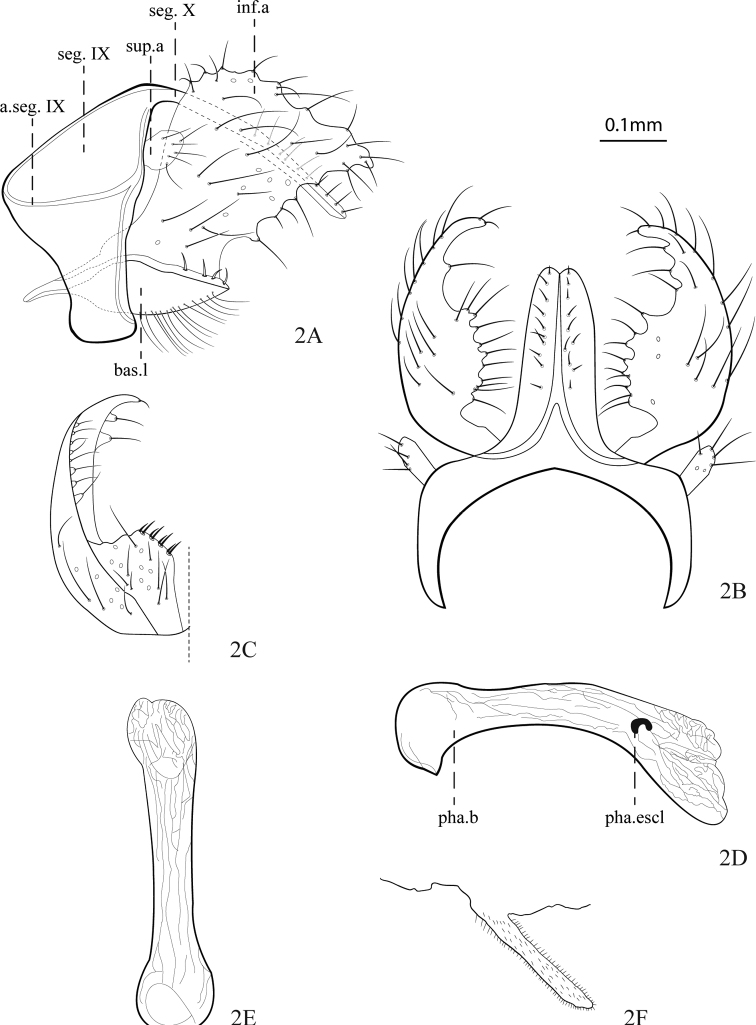
*Helicopsycheralphi* sp. nov., male **A** genitalia, lateral view **B** segments IX and X and inferior appendages, dorsal view **C** inferior appendage, ventral view **D** phallus, lateral view **E** phallus, ventral view **F** sternum VI, lateral view. Abbreviations: seg. IX = abdominal segment IX; a.seg. IX = apodeme of abdominal segment IX; sup.a = superior appendage; seg. X = abdominal segment X; bas.l = basomesal lobe; inf.a = inferior appendage; pha.b = phallobase; pha.scl. = phallotremal sclerite.

**Figure 3.**
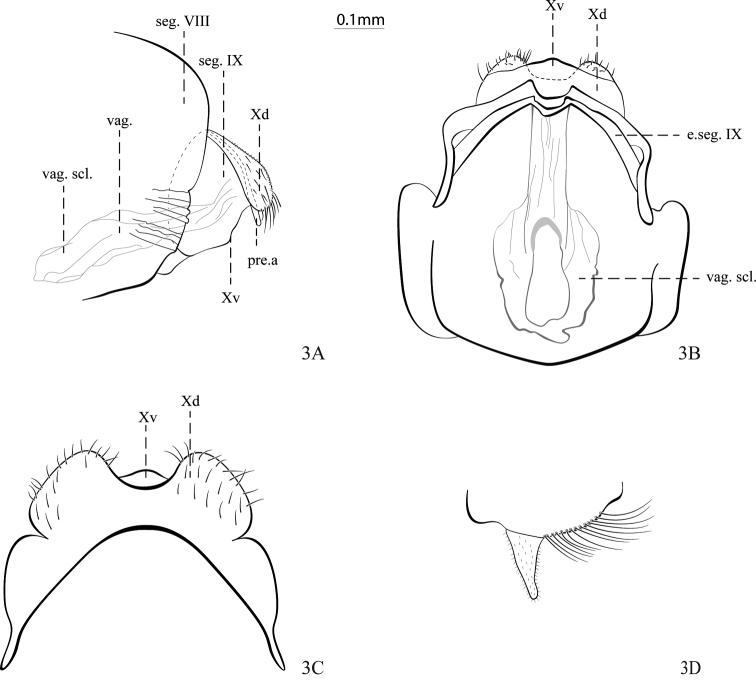
*Helicopsycheralphi* sp. nov., female **A** genitalia, lateral view **B** genitalia, ventral view **C** genitalia, dorsal view **D** sternum VI, lateral view. Abbreviations: seg. VIII = abdominal segment VIII; seg. IX = abdominal segment IX; e.seg. IX = external part of abdominal segment IX; pre.a = preanal appendage; Xd = dorsal branch abdominal segment X; Xv = ventral branch abdominal segment X; vag. = vagina; vag. scl. = vaginal sclerite.

**Figure 4.**
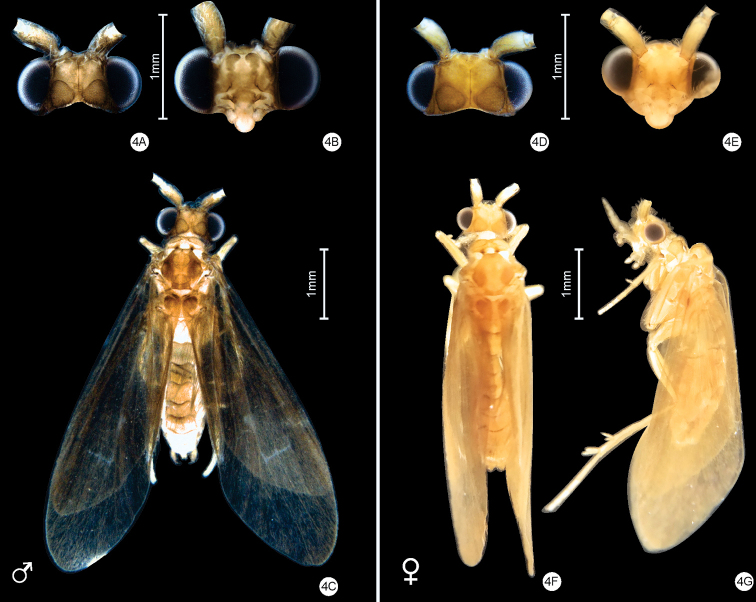
*Helicopsycheralphi* sp. nov., adult **A** male head, dorsal view **B** male head, frontal view **C** male habitus, dorsal view **D** female head, dorsal view **E** male head, frontal view **F** female habitus, dorsal view **G** female habitus, lateral view.

**Figure 5.**
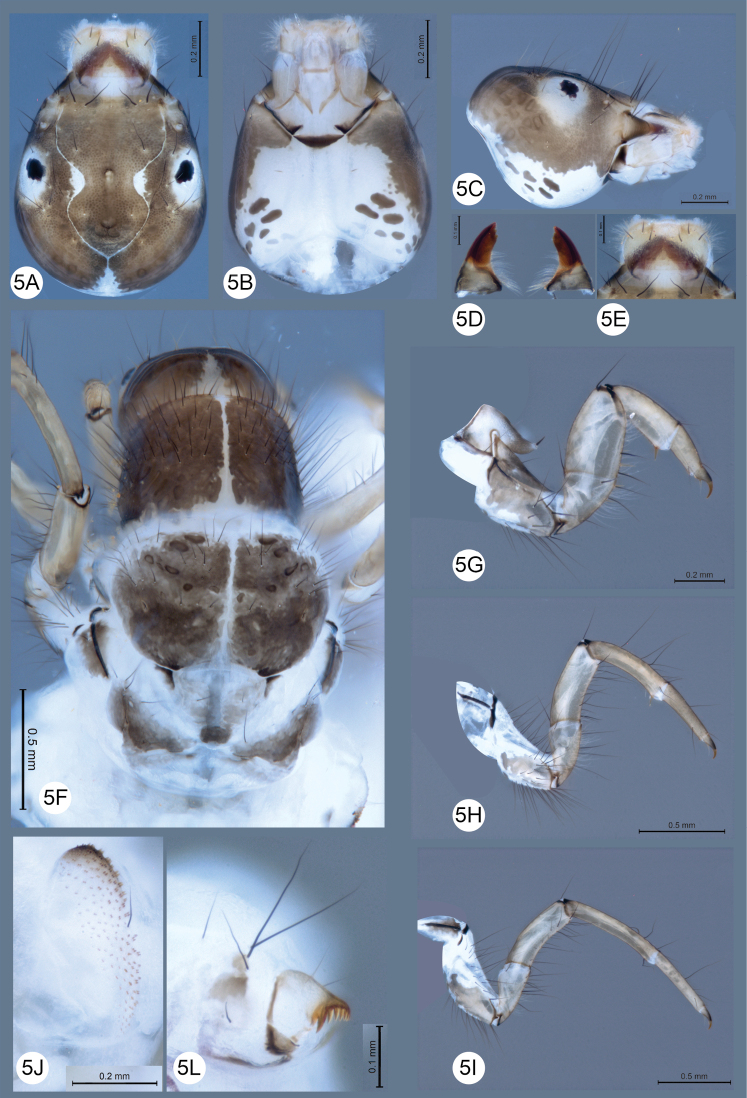
*Helicopsycheralphi* sp. nov., larvae **A** head, dorsal view **B** head, ventral view **C** head, left lateral view **D** mandibles, dorsal view **E** labrum, dorsal view **F** notos, dorsal view **G** proleg, lateral view **H** mesoleg, lateral view **I** metaleg, lateral view **J** lateral hump **L** anal legs.

**Figure 6.**
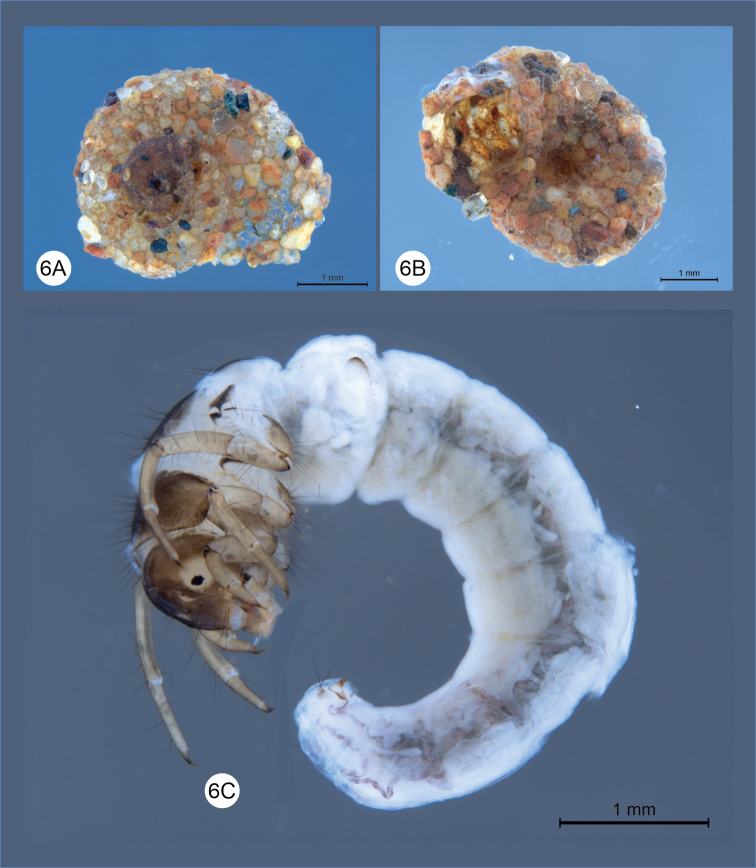
*Helicopsycheralphi* sp. nov., larvae and case **A** larval case, dorsal view **B** larval case, ventral view **C** larval lateral habitus.

**Figure 7.**
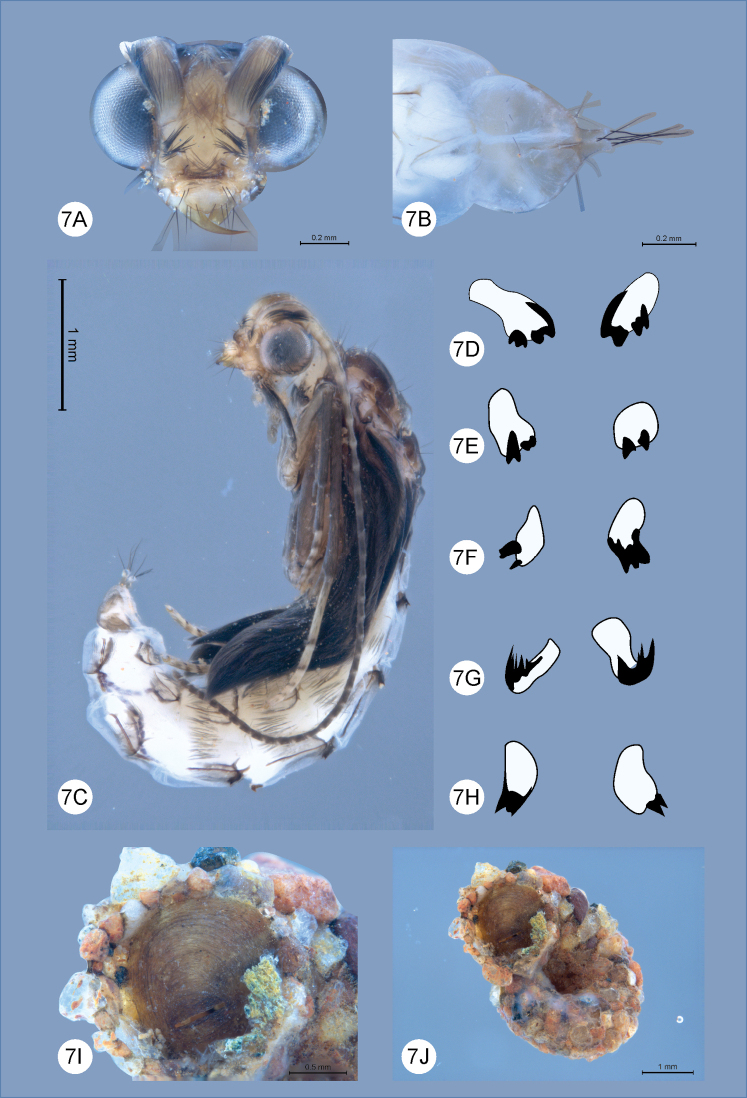
*Helicopsycheralphi* sp. nov., pupa and case **A** pupa front **B** abdominal segment IX and anal processes, dorsal view **C** pupa lateral habitus **D–H** abdominal segments I–V, dorsal, with details of dorsal hook plates **I** pupa case, ventral view **J** pupa case with sieve membrane highlighted, ventral view.

Figs 2 , 3 , 4 , 5 , 6 , 7

#### Material examined.

***Holotype*.** Brazil, 1 male; Pernambuco, Triunfo, Pinga stream; 7°52'3"S, 38°7'13"W, el. 890 m; 21.ix.2017; Cavalcante-Silva, A. leg.; UV light pan trap (MZSP). ***Paratypes*.** Same data as holotype, except 5 males; Grito stream; 7°51'41"S, 38°5'25"W, el. 740 m; 09.viii.2018 (UFBA); same except 3 males; Laje stream; 7°52'13"S, 38°5'18"W, el. 580 m; 07.viii.2018 (DZRJ); same except 6 males; Pinga stream; 7°52'3"S, 38°7'13"W, el. 890 m; 21.ix.2017 (MZSP); same except 6 males (INPA); same except 1 female; 7°52'3"S, 38°7'13"W, el. 890 m; 03.ii.2019 (MZSP); same except 5 females; 7°52'5,5"S, 38°7'15,6"W, el. 870 m; 01.v.2019; Cavalcante-Silva, A, Pereira, R. leg. (MZSP); same except 6 females; 7°52'5,5"S, 38°7'15,7"W, el. 865 m; 01.v.2019 (UFBA); same except 6 females (DZRJ); same except 6 females (INPA).

#### Diagnosis.

The new species is distinguished from all other congeners by the following characters of the male genitalia: inferior appendage subtriangular, acuminated in posterior region, basomesal lobe subtriangular ~ 1/2 the length of the inferior appendage, in lateral view, trapezoid, with spine-like setae in posterior margin, in ventral view; abdominal segment X slender, slightly cleft at the apex, in dorsal view. The characters of the genitalia of new species are morphologically similar to *Helicopsycheflinti*Johanson (1999). The new species presents abdominal segment X with a rounded apex, and a medial row of spine-like setae, in dorsal view (while *H.flinti* presents abdominal segment X with apex nearly straight, side row of spine-like setae), and inferior appendage with strongly projecting mesal margin, forming a large, rounded lobe, in dorsal view (while *H.flinti* presents an inferior appendage without a large mesal lobe).

#### Description.

**Adults** (Fig. 4): length of forewing 4.1–5.2 mm (*n* = 20). Wings: forewing without discoidal cell, without medial cell, with thyridial cells; hind wing without discoidal cell, without thyridial cell. Head: brownish; antennae yellowish, shorter than forewing length, scape yellowish, shorter than head length, covered with long setae (Fig. 4E–G). Thorax: pronotum brownish, with warts, filiform, covered with small and ferruginous setae; mesoscutum brownish, with mesoscutal warts spherical and not covered with setae; mesoscutellum brownish, with mesoscutellar warts spherical and not covered with setae (Fig. 4G); legs yellowish, tibial spur formula 2,2,4.

**Male** (Figs 2, 4A–C): body length ~ 3.3–4.6 mm (*n* = 20).

***Head***: interantennal warts present, brownish, spherical, covered with small setae; posteroantennal warts present, brownish, club shaped, covered with long setae; cephalic warts present, brownish, subtriangular, covered with long setae; postocular warts present, filiform, brownish, covered with long setae (Fig. 4A–C); maxillary palps yellowish, with two segments, covered with long ferruginous setae; labial palps yellowish, with three segments, covered with long ferruginous setae. Abdomen: abdominal sternum VI process present, almost same length as segment, tubular, apically rounded, covered with small microtrichiae (Fig. 2F).

***Genitalia*.** Abdominal segment IX with slightly concave anteroventral margin in ventral half; apodeme well developed laterally, located midlaterally on segment; posterior margin nearly straight, in lateral view (Fig. 2A), anterior margin strongly concave, in dorsal view (Fig. 2B); preanal appendages setose, rounded in lateral view (Fig. 2A), clavate in dorsal view (Fig. 2B). Abdominal segment X tubular, dorsal margin slightly curved, in lateral view (Fig. 2A); slender, mesodorsal borders inverted Y-shaped, bearing two rows of short setae, near the center, in dorsal view (Fig. 2B). Inferior appendage subtriangular, acuminated in posterior region, in lateral view (Fig. 2A); anterior margin slightly convex, posterior margin undulated and tapered apex, in dorsal view (Fig. 2B); basomesal lobe of inferior appendage, in lateral view well developed, with ventral margin covered with long setae and dorsal margin with spine-like setae, in ventral view (Fig. 2C). Phallus tubular, phallobase rounded, ventral view (Fig. 2E), acuminate at anteroventral border, in lateral view (Fig. 2D), slightly down curved; phallotremal sclerite conspicuous, moon shaped in lateral view (Fig. 2D).

**Female** (Figs 3, 4D–G): body length ~ 3.9–5.4 mm (*n* = 20).

***Head***: interantennal warts present, brownish, spherical, covered with small setae; postero-antennal warts present, brownish, covered with long setae; cephalic warts present, brownish, subtriangular, covered with long setae (Fig. 4D–F); postocular warts present, filiform, brownish, covered with long setae (Fig. 4F); maxillary palps yellowish, with 5-segments, covered with long and yellowish setae; labial palps yellowish, with 3-segments, covered with long yellowish setae (Fig. 4G). Abdomen: abdominal sternum VI process present, ~ 1/3 segment length, tubular and apically rounded, covered with small microtrichiae (Fig. 3D).

***Genitalia*.** Abdominal segment IX is well separated from abdominal segment VIII and indistinctly separated from abdominal segment X, anterior margin convex, in lateral view (Fig. 3A); external part of abdominal segment IX apically incised, in ventral view (Fig. 3B). Preanal appendage long and filiform, in lateral view (Fig. 3A). Abdominal segment X with two branches; dorsal branch narrow, base with apex broad, rounded and covered with long setae, in lateral view (Fig. 3A), bilobed with U-shaped with apical incision, in dorsal view (Fig. 3C); ventral branch with sinuous margin, in lateral view (Fig. 3A), and apex obtuse in ventral and dorsal view (Fig. 3B, C). Vagina with thick anterior margin, in ventral view (Fig. 3B); vaginal sclerite slender along its length, in lateral view (Fig. 3A), finger-shaped projection on the anterior margin, internal sclerite long, with sclerotized lateral margins, in ventral view (Fig. 3B).

**Larva (5^th^ instar)** (Figs 5A–L, 6C): Body total length 2.9–3.6 mm (*n* = 10).

***Head***: oval, with anterior margin 1.3 × broader than posterior margin, in dorsal view (Fig. 5A), mostly light brown, with pale region on anterolateral margin of the head capsule until antenna region, around stemmata, medial region of frontoclypeus margin, and posteromedial region of head capsule, in dorsal view (Fig. 5A), with lateral region light brown, in ventral view (Fig. 5B), cardo and anterior ventral apotome dark brown, six and eight brown muscle scars in left and right, respectively, in dorsal view (Fig. 5A), head capsule with muscle scar light brown in posterolateral region, in dorsal view (Fig. 5A), with frontal area flattened, muscle scars in basal region, cardo and ventral apotome sclerotized, ventral view (Fig. 5B); with muscle scar light brown in posterior region, in lateral view (Fig. 5C); frontoclypeus and adjacent areas nearly flat and margined with semicircular carina, frontoclypeal suture with strongly delimited margin, frontoclypeal with one muscle scar brown in medial region, and three in posterior region, in dorsal view (Fig. 5A); labrum translucent, with short setae covering the anterior margin, in ventral view (Fig. 5B); mandibles asymmetrical, each internal margin with pale, long, thin setae; left mandible with three teeth, the apical tooth trilobed, mesal and basal tooth acute; right mandible with three teeth, the apical tooth trilobed, mesal and basal tooth obtuse, in dorsal view (Fig. 5D–E); chaetotaxy of head as in Figure 5A–E. Thorax: pronotum brown with dark muscle scars, anterior region with row of long setae at margin, covered long setae to near medial region, posterior margin sinuous and lighter with few and scattered setae, in dorsal view (Fig. 5F), trochantin almost as long as foreleg coxae, finger shaped with one spinelike setae in apex (Fig. 5G); mesonotum lighter than pronotum, with pale regions in medial region, muscle scars in dark brown shades, four pairs in anterior region and one pair in posterior region, posterior margins angulate, in dorsal view (Fig. 5F); metanotum with three pairs of sclerites, two pairs of anteromesal (sa1) sclerites small, one pairs anterior subtriangular, and one pairs irregular bearing one setae and one pair of posterior subtriangular sclerites (fused sa2 and sa3 sclerites), each bearing single seta posteromesally (sa2) and several setae anterolateral (sa3), in dorsal view (Fig. 5F); lateral hump oval, apical region mostly dark, one short setae in anteromedial region (Fig. 5J); thoracic legs with chaetotaxy as in Figure 5G–I; The foreleg has a length equivalent to 2/3 of the midleg and 1/2 of the hindleg, foreleg segments robust and short, mid and hind leg segments filiform and long (Fig. 5I). Abdomen: anal prolegs each with lateral sclerites curved, anal claw elongates, with accessory parallel teeth pectinate, arranged like comb (Fig. 5L).

***Larval case*** (Figs 6A–B, 7I–J): length 2.9–3.6 mm (*n* = 10). Made with cemented sand grains, forming a snail–like, helical case, case with two 1/2 whorls at the end of the phase, with umbilicus open and deep.

***Pupa*** (Fig. 7A–J): body length 3.3–4.1 mm (*n* = 10). Generally dark brown, almost black, with yellowish abdomen; Head: mandibles curved with wide bases, each with length 2.5 × basal width, apex pointed and internal margin smooth (Fig. 7A). Abdomen: paired anterior dorsal hook plates on segments II–V, pair of posterior dorsal hook plates on segment IV asymmetrical; general morphology of dorsal hook plates as in (Fig. 5C–H); terminal abdominal segment rounded, with two divergent digitate processes, each process bearing one subapical and three apical setae (Fig. 7B).

#### Etymology.

This species is named in honor of Dr. Ralph W. Holzenthal, for his outstanding contribution to the study of Neotropical caddisflies, and also as an acknowledgment for collaboration and his contributing to the training of young researchers.

#### Material additional.

A1 (1 female), C6 (643 males); C7 (10 larvae, 10 pupae); D2 (34 males); D7 (1 male); D17 (14 males); D18 (379 males); D18 (49 females); D20 (32 females); D22 (1 larvae); E1 (1 male); F2 (30 males); F3 (17 males) (UFBA) (Table 1).

#### Distribution.

Brazil (Pernambuco state).

##### ﻿Caddisflies from Brejo de Altitude de Triunfo

###### HYDROPSYCHIDAE

### ﻿Key to Brazilian species of Helicopsyche (Feropsyche), except *H.braziliensis* (Swainson, 1840) and *H.helicoidella* (Vallot, 1855)

*Helicopsychebraziliensis* (Swainson, 1840) and *H.helicoidella* (Vallot, 1855) are not included in the key because their males are not known.

**Table:** 

1	Inferior appendage with distal region rounded, in lateral view (Dumas and Nessimian 2019: fig. 2A)	**2**
–	Inferior appendage with distal region acuminated, in lateral view (Johanson 2002: fig. 21A)	**11**
2	Abdominal segment X with projections (Dumas and Nessimian 2019: fig. 2A)	**3**
–	Abdominal segment X without projections (Holzenthal et al. 2016: fig. 2B)	**6**
3	Abdominal segment IX with anterior lobe rounded, anterodorsal margin notched, in lateral view (Dumas and Nessimian 2019: fig. 4A); abdominal segment X with apical cleft V-shaped, in dorsal view (Dumas and Nessimian 2019: fig. 8B)	**4**
–	Abdominal segment IX with anterior lobe acuminate, anterodorsal margin nearly straight, in lateral view (Dumas and Nessimian 2019: fig. 1A); abdominal segment X with apical cleft U-shaped, in dorsal view (Holzenthal et al. 2016: fig. 2B)	**5**
4	Abdominal segment X rectangular with projections less developed, in dorsal view (Dumas and Nessimian 2019: fig. 4B); inferior appendage with rounded apex and without projections, in ventral view (Dumas and Nessimian 2019: fig. 4C)	***H.luziae* Dumas & Nessimian, 2019**
–	Abdominal segment X deltoid with pair of large tab-like midlength projections, in dorsal view (Dumas and Nessimian 2019: fig. 8B); inferior appendage with acuminate apex and with finger shaped projection, in ventral view (Dumas and Nessimian 2019: fig. 8C)	***H.planorboides* Machado, 1957**
5	Inferior appendage with length equal to or shorter than abdominal segment X, basal lobe nearly as wide as distal lobe, in lateral view (Dumas and Nessimian 2019: fig. 1A); basomesal lobe trapezoid and unprojected, in lateral view (Dumas and Nessimian 2019: fig. 1A)	***H.bendego* Dumas & Nessimian, 2019**
–	Inferior appendage longer than abdominal segment X, basal lobe narrower than distal lobe, in lateral view (Holzenthal et al. 2016: fig. 2A); basomesal lobe finger shaped and projected, in lateral view (Holzenthal et al. 2016: fig. 2A)	***H.guara* Holzenthal, Blahnik & Calor, 2016**
6	Inferior appendage longer than abdominal segment X, distal lobe narrow and longer, in lateral view (Johanson 2002: figs 8D, 11D)	**7**
–	Inferior appendage with length equal to or shorter than abdominal segment X, distal lobe wide and short, in lateral view (Holzenthal et al. 2016: fig. 1A)	**8**
7	Abdominal segment X with a row of setae going from base to the apex, in dorsal view (Johanson 2002: fig. 8E); inferior appendage shorter than abdominal segment X, in dorsal view (Johanson 2002: fig. 8E)	***H.vergelana* Ross, 1956**
–	Abdominal segment X with a row of setae going from middle to the apex, in dorsal view (Johanson 2002: fig. 11E); inferior appendage equal or longer than abdominal segment X, in dorsal view (Johanson 2002: fig. 11E)	***H.tapadas* Denning, 1966**
8	Abdominal segment X with apical cleft, in dorsal view (Holzenthal et al. 2016: fig. 1B); inferior appendage with base and apex with subequal width, in ventral view (Holzenthal et al. 2016: fig. 1C)	**9**
–	Abdominal segment X without apical cleft, in dorsal view (Dumas and Nessimian 2019: fig. 2B); inferior appendage with wide base and apex without subequal width, in ventral view (Silva et al. 2014: fig. 1B)	**10**
9	Inferior appendages bear very prominent spine-like setae on their apicomesal face and mesally at midlenght, in ventral view (Holzenthal et al. 2016: fig. 1C); basomesal lobe oval and short, in ventral view (Holzenthal et al. 2016: fig. 1C)	***H.angeloi* Holzenthal, Blahnik & Calor, 2016**
–	Inferior appendages without setae on their apicomesal face and mesally at midlenght, in ventral view (Johanson and Malm 2006: fig. 49); basomesal lobe finger-shaped and ~ 1/2 the length of the inferior appendage, in ventral view (Johanson and Malm 2006: fig. 49)	***H.cipoensis* Johanson & Malm, 2006**
10	Abdominal segment X with apical cleft V-shaped, in dorsal view (Dumas and Nessimian 2019: fig. 2B); inferior appendages with half the length of the abdominal segment X, and with well-developed and strongly rounded protuberance, in dorsal view (Dumas and Nessimian 2019: fig. 2B)	***H.daome* Dumas & Nessimian, 2019**
–	Abdominal segment X with deep and short apical cleft U-shaped, in dorsal view (Silva et al. 2014: fig. 1A); inferior appendages with subequal length than abdominal segment X, and without rounded protuberance (Silva et al. 2014: fig. 1B)	***H.timbira* Silva, Santos & Nessimian, 2014**
11	Basomesal lobe not or very little projected on anterobasal margin of the inferior appendage, in lateral view (Gama-Neto et al. 2019: fig. 3C)	**12**
–	Basomesal lobe well projected on anterobasal margin of the inferior appendage, in lateral view (Gama-Neto et al. 2019: fig. 2C)	**21**
12	Abdominal segment X with projections (Holzenthal et al. 2016: fig. 3B)	**13**
–	Abdominal segment X without projections	**14**
13	Inferior appendage deltoid, in lateral view; basomesal lobe unprojected, in ventral view (Holzenthal et al. 2016: fig. 3C)	***H.lazzariae* Holzenthal, Blahnik & Calor, 2016**
–	Inferior appendage globose with distal finger shaped projection and ventromedial setose projection, in lateral view (Gama-Neto et al. 2019: fig. 3D); basomesal lobe triangular and well projected, in ventral view	***H.inflata* Gama-Neto, Ribeiro & Passos, 2019**
14	Abdominal segment X subretangular with apex nearly straight, in dorsal view (Johanson 2002: fig. 46E)	**15**
–	Abdominal segment X ovaled with apex rounded, in dorsal view (Dumas and Nessimian 2019: fig. 3B)	**16**
15	Abdominal segment IX with broad base, in lateral view (Dumas and Nessimian 2019: fig. 5A); abdominal segment X with acuminate apex, in lateral view (Dumas and Nessimian 2019: fig. 5A), and without apical cleft, in dorsal view (Dumas and Nessimian 2019: fig. 5B)	***H.petri* Dumas & Nessimian, 2019**
–	Abdominal segment IX with short base, in lateral view; abdominal segment X with rounded apex, in lateral view (Johanson 2002: fig. 46D), and with shallow, short apical cleft, in dorsal view (Johanson 2002: fig. 46E)	***H.monda* Flint, 1983**
16	Inferior appendage with a wide concavity in the posterobasal margin; basomesal lobe square with almost straight margins, in ventral view (Johanson 2002: fig. 45F)	**17**
–	Inferior appendage nearly straight or with a short convexity in posterobasal margin; basomesal lobe finger shaped with rounded margins, in ventral view (Dumas and Nessimian 2019: fig. 6C)	**19**
17	Abdominal segment X with lateral margin nearly straight, subapical cluster of setae and apex with a deep, and long cleft, in dorsal view (Johanson and Holzenthal 2004: fig. 18)	***H.succincta* Johanson & Holzenthal, 2004**
–	Abdominal segment X with lateral margin convex, with a row of setae going from base to the apex, and apical shallow, short cleft, in dorsal view (Vilarino and Calor 2017: fig. 4B)	**18**
18	Inferior appendage subrectangular, with a large lobe inner face, in dorsal view, (Johanson 2002: fig. 45E); basomesal lobe wide and ~ 1/2 the length of the inferior appendage, in ventral view (Johanson 2002: fig. 45F)	***H* . *valligera* Flint, 1983**
–	Inferior appendage in boomerang shape, without large lobe inner face, in dorsal view (Vilarino and Calor 2017: fig. 18); basomesal lobe very short of the length of the inferior appendage, in ventral view (Vilarino and Calor 2017: fig. 4D)	***H.guariru* Vilarino & Calor, 2017**
19	Inferior appendage subrectangular with wide basal lobe almost as wide as distal lobe, in lateral view (Johanson 2002: fig. 21A); basomesal lobe ~ 1/2 the length of the inferior appendage, in ventral view (Johanson 2002: fig. 21B)	***H* . *muelleri* Banks, 1920**
–	Inferior appendage subtriangular, basal lobe narrow and distal lobe wide; basomesal lobe less than half the length of the inferior appendage, in ventral view (Dumas and Nessimian 2019: fig. 3C)	**20**
20	Abdominal segment X and Inferior appendages subequal in length, with clusters setae on apex, in dorsal view (Dumas and Nessimian 2019: fig. 6B); inferior appendage deltoid with distal finger shaped projection, in lateral view (Dumas and Nessimian 2019: fig. 6A); basomesal lobe globose and bifid, (Dumas and Nessimian 2019: fig. 6C)	***H.shaamunensu* Dumas & Nessimian, 2019**
–	Abdominal segment X shorter than inferior appendage in length, with a row of setae going from base to the apex, in dorsal view (Dumas and Nessimian 2019: fig. 3D); inferior appendage triangular with distal lobe long with acuminated apex, in lateral view (Dumas and Nessimian 2019: fig. 3A); basomesal lobe finger shaped, in ventral view (Dumas and Nessimian 2019: fig. 3C)	***H.dinoprata* Dumas & Nessimian, 2019**
21	Basomesal lobe filiform shaped with a cluster of spine-like setae in distal region, in lateral view (Souza et al. 2017: fig. 1A), apex rounded covered with spine-like setae, in ventral view (Gama-Neto et al. 2019: fig. 2D)	**22**
–	Basomesal lobe subtriangular with a cluster of spine-like setae in dorsal and ventral margin, in lateral view (Fig. 2A), apex nearly straight covered with spine-like setae, in ventral view (Fig. 2C)	**23**
22	Abdominal segment IX with anterior lobe acuminated, in lateral view (Souza et al. 2017: fig. 1A); abdominal segment X rectangular, row of setae going from base to the apex, apex nearly straight with shallow and short cleft, in dorsal view (Souza et al. 2017: fig. 1D); inferior appendage with nearly straight posterior margin and with setose projection and shorter apicodorsal projection, in lateral view (Souza et al. 2017: fig. 1A)	***H.catoles* Souza, Gomes & Calor, 2017**
–	Abdominal segment IX with anterior lobe rounded, in lateral view (Gama-Neto et al. 2019: fig. 2C); abdominal segment X oval, subapical cluster of setae, apex rounded without cleft, in dorsal view (Gama-Neto et al. 2019: fig. 2E); inferior appendage with wide concavity on posterior margin and without setose projection and longer apicodorsal projection, in lateral view (Gama-Neto et al. 2019: fig. 2C)	***H.carajas* Gama-Neto, Ribeiro & Passos, 2019**
23	Abdominal segment IX with anterodorsal margin notched, in lateral view (Johanson and Malm 2006: fig. 29); abdominal segment X with shallow and long cleft, in dorsal view (Johanson and Malm 2006: fig. 30); inferior appendage truncated with apical tooth, in ventral view; basomesal lobe short and subtriangular, in ventral view (Johanson and Malm 2006: fig. 31)	***H.paprockii* Johanson & Malm, 2006**
–	Abdominal segment IX with anterodorsal margin nearly straight, in lateral view (Fig. 2A); abdominal segment X with short or without cleft, in dorsal view; inferior appendage with apical projection finger shaped, in ventral view (Fig. 2C); basomesal lobe wide and trapezoid, ventral view (Fig. 2C)	**24**
24	Abdominal segment X with apex rounded, medial row of spine-like setae, in dorsal view (Fig. 2B); inferior appendage with inner margin strongly projected mesad, forming a rounded large lobe, in dorsal view (Fig. 2B)	***H.ralphi* sp. nov.**
–	Abdominal segment X with apex nearly straight, side row of spine-like setae, in dorsal view (Johanson 1999: fig. 4); inferior appendage without large lobe inner face, in dorsal view (Johanson 1999: fig. 4)	***H.flinti* Johanson, 1999**

### ﻿ Smicridea (Smicridea) palifera

Taxon classificationAnimaliaTrichopteraHelicopsychidae

Flint

90F7E2C9-D7DC-548B-A380-B373164532BE

Smicridea (Smicridea) palifera Flint, 1981: 23 [type locality: Venezuela, Aragua, Maracay, El Limón; NMNH; male; female].

#### Material examined.

Brazil: Pernambuco: B2 (71 males, 9 females); C1 (4 males, 2 females); C5 (4 males); C6 (2 males, 3 females); D4 (1 female); D5 (1 female); D6 (4 males, 3 females); D7 (27 males, 15 females); D10 (1 female); D11 (3 males, 1 female); D15 (1 male); D17 (1 male); D18 (3 males); D19 (3 males, 2 females); D20 (6 males, 10 females); F1 (1 female).

#### Distribution.

Brazil (AL, ES, MA, MT, MG, PB, PE, RJ, RO), Grenada, and Venezuela.

#### Remarks.

This species differs from all other species in the *Smicrideanigripennis* group due to the presence of a simple aedeagus, with only a sclerotized spine, and large rounded lobe in segment X (Flint 1981). Smicridea (Smicridea) palifera presents a wide distribution in Brazil, except in the south of the country (Santos et al. 2022). In the Northeast region it has been registered in four states (altitude range of 53 m and 814 m) (Souza et al. 2013a; Desidério et al. 2017; Desidério et al. 2020). It was recorded in the Caatinga domain, Pernambuco state (Souza et al. 2013a) and later the Cerrado and Atlantic Forest domains (Desidério et al. 2017; Desidério et al. 2020). This is the first record in *Brejo de Altitude*.

##### LEPTOCERIDAE

### ﻿ Oecetis excisa

Taxon classificationAnimaliaTrichopteraHelicopsychidae

Ulmer

1C48BCBD-4AAE-58BE-9078-FA41EFF65F34

Oecetis excisa Ulmer, 1907: 15 [type locality: Argentina, Chaco de Santa Fé, Las Garzas, Río Las Garzas, 25 km W Ocampo; MNHNP; male].

#### Material examined.

Brazil: Pernambuco: B2 (1 male, 1 female); B3 (1 male); C1 (2 males, 8 females); C3 (1 male); C5 (1 male); D3 (17 males); D5 (1 female); D9 (20 females); D10 (1 male); D11 (1 male, 2 females); D12 (16 males, 4 females); D21 (1 female); E1 (1 male).

#### Distribution.

Argentina, Bolivia, Brazil (BA, CE, GO, MS, MT, PA, PB, PE, RN, SP), Mexico, Paraguay, and Venezuela.

#### Remarks.

The examined specimens match the description of Ulmer (i.e., tibial spur formula 1,2,2), unlike specimens examined by Quinteiro and Calor (2015), for states of Bahia, Mato Grosso, Paraíba, and Rio Grande do Norte, which presented tibial spur formula 0,2,2. This species is widely distributed in Brazil, including several records in the Northeast region (Quinteiro and Calor 2015; Desidério et al. 2017). Souza et al. (2013a) recorded the occurrence of the species in the Caatinga domain, Amaraji municipality, Pernambuco state (altitude 320 m). This study provides the first record of species in *Brejo de Altitude*.

##### PHILOPOTAMIDAE

### ﻿ Chimarra potiguar

Taxon classificationAnimaliaTrichopteraHelicopsychidae

Queiroz, Dias & Calor

2A8972C6-6371-5FCC-B388-0E941915784A

Chimarra potiguar Queiroz, Dias & Calor, 2020: 101 [type locality: Brazil, Rio Grande do Norte, Portalegre, Pinga Stream, MZUSP; male].

#### Material examined.

Brazil: Pernambuco: C1 (4 males, 17 females); C2 (1 male); C4 (2 males); C5 (5 males); C6 (7 males, 4 females); D1 (3 males); D2 (54 males, 55 females); D7 (6 males, 7 females); D10 (6 males, 5 females); D11 (2 males, 2 females); D12 (2 females); D13 (1 female); D14 (2 females); D16 (1 male, 3 females); D17 (2 males, 2 females); D18 (39 males, 18 females); D19 (28 males, 10 females); D20 (52 males, 42 females); D21 (4 males, 3 females); E1 (11 females); E2 (2 females).

#### Distribution.

Brazil (RN, PE [new record]).

#### Remarks.

The occurrence of this species was recorded only for the Brejo de Altitude de Portalegre, Rio Grande do Norte state (altitude of 642 m) (Queiroz et al. 2020). The record in Brejo de Altitude de Triunfo (altitude range 580–940 m) is also the first record for Pernambuco state.

##### POLYCENTROPODIDAE

### ﻿ Cyrnellus fraternus

Taxon classificationAnimaliaTrichopteraHelicopsychidae

(Banks)

2896C64C-EC7F-53AC-994B-9DDACF4BC4A3

Cyrnus fraternus (Banks, 1905): 17 [type locality: United States, Maryland, Plummer’s Island; MCZ; female].

#### Material examined.

**Brazil: Pernambuco**: C3 (1 male); D2 (4 males); D7 (1 male); D11 (3 males); D12 (4 males); D13 (4 males); D14 (7 males); D16 (8 males); D17 (1 male); D21 (1 male).

#### Distribution.

Argentina, Brazil (AM, BA, ES, MA, MG, MS, MT, PA, PE [new record], PI, PR, RJ, SC), Costa Rica, El Salvador, Ecuador, Mexico, Nicaragua, Panama, Paraguay, Suriname, United States, Uruguay, and Venezuela.

#### Remarks.

*Cyrnellus* Banks contains 12 species in the Neotropical region, and is widely distributed in North, Central, and South America (Morse 2022). *Cyrnellusfraternus* has a distribution from the USA to Argentina, being the most widely distributed caddisfly on the continent (see Holzenthal and Calor 2017). Currently, it has a known distribution in several regions of Brazil and in the Northeast is registered for the Caatinga and Cerrado domains (Dumas et al. 2010; Takiya et al. 2016; Desidério et al. 2017). In this study the species distribution is extended, representing the first record for the state of Pernambuco.

### ﻿ Cyrnellus mammillatus

Taxon classificationAnimaliaTrichopteraHelicopsychidae

Flint

ABFB22FE-4882-50E7-A056-A59075759874

Cyrnellus mammillatus Flint, 1971: 30 [type locality: Brazil [Edo. Amazonas], Lago des Rio Luna am oberen Teil; NMNH; male].

#### Material examined.

**Brazil: Pernambuco**: B2 (6 males); D12 (3 males).

#### Distribution.

Argentina, Brazil (AM, MA, MG, MS, PA, PE, PI, PR, RJ, SP), Ecuador, Paraguay, Peru, and Uruguay.

#### Remarks.

In the Northeast of Brazil, the occurrence of this species was recorded for the Caatinga and Cerrado domains (altitude range 60–448 m) (Souza et al. 2013a; Desidério et al. 2017; Moreno et al. 2020). This study expands its occurrence for rainforest islands of higher elevations (altitude range 580–940 m) on Brejo de Altitude.

### ﻿ Cyrnellus kozepes

Taxon classificationAnimaliaTrichopteraHelicopsychidae

Oláh

D37499A2-3CBA-5BD8-BE0B-BA3EEB46FCEC

Cyrnellus kozepes Oláh, 2016: 159 [type locality: Argentina, Corientes Province, Carlos Pellegrini Posada, Aguape, 28°32'26"S, 57°10'20"W; male].

#### Material examined.

**Brazil: Pernambuco**: D14 (1 male).

#### Distribution.

Argentina and Brazil (PE [new record]).

#### Remarks.

Previously recorded only for Argentina (type locality) (Oláh 2016). This study extends the known distribution of this species and provides the first record for Brazil. This disjunct distribution may be the result of omission errors, the article describing the species is difficult to access and the illustration provided lacks details. In order to avoid future the errors, omission we provide an illustration richer in details.

##### HYDROPTILIDAE

### ﻿ Metrichia peluda

Taxon classificationAnimaliaTrichopteraHelicopsychidae

Santos, Takiya & Nessimian

21211E85-A301-5313-A4EA-60130F8C1F76

Metrichia peluda Santos, Takiya & Nessimian, 2016: 35 [type locality: Brazil, Rio de Janeiro, Itatiaia, 1^st^ order tributary of Rio Palmital, 22°25'40"S, 44°32'46"W, el. 584 m; DZRJ; male].

#### Material examined.

**Brazil: Pernambuco**: D3 (7 males); D7 (1 male).

#### Distribution.

Brazil (PE [new record], RJ).

#### Remarks.

Previously recorded only from the type locality, domain of the Atlantic Forest (Southeast region of Brazil), the known distribution of this species is extended into the Northeast region with this study.

### ﻿ Neotrichia feolai

Taxon classificationAnimaliaTrichopteraHelicopsychidae

Santos & Nessimian

07C8C7B7-3F79-5BE6-9421-7B2D0820AFB3

Neotrichia feolai Santos & Nessimian, 2009: 766 [type locality: Brazil, Amazonas, Rio Preto da Eva (tributary to Rio Preto da Eva, 02°38'14,6"S, 59°44'09,9"W); INPA; male].

#### Material examined.

**Brazil: Pernambuco**: D3 (2 males).

#### Distribution.

Brazil (AM, PE) and Venezuela.

#### Remarks.

This species was previously recorded only for the Amazon rainforest (Northern region of Brazil) and Venezuela (Santos et al. 2022). Subsequently, Souza et al. (2013b) recorded it for the Caatinga, Northeast Brazil. This study reports this species for the first time in Atlantic Forest (Brejo de Altitude).

### ﻿ Oxyethira tica

Taxon classificationAnimaliaTrichopteraHelicopsychidae

Holzenthal & Harris

2568FA26-007B-5E95-A156-857F79FE1D2C

Oxyethira tica Holzenthal & Harris, 1992: 168 [type locality: Costa Rica, Guanacaste, Parque Nacional Santa Rosa, Quebrada El Duende near La Casona, 10.838°N, 85.614°W; NMNH; male; female].

#### Material examined.

**Brazil: Pernambuco**: B2 (9 males); C1 (2 males); D3 (32 males); D7 (2 males).

#### Distribution.

Brazil (AL, AM, BA, CE, MA, MG, PB, PE, PI, RJ, SE), Costa Rica, Dominica, Ecuador, French Guiana, Grenada, Guadeloupe, Honduras, Martinique, Mexico, Nicaragua, Panama, St. Lucia, St. Vincent, Trinidad, and Venezuela.

#### Remarks.

Holzenthal and Harris’ (1992) description matches the specimens examined, except for the number of segments in the antennae (33 segments) in the specimens observed. Previously reported for the North and Southeast regions of Brazil Takiya et al. (2016) recorded the presence of the species for the Northeast region, Caatinga domain (Ceará state) and Souza and Santos (2017) extended the distribution for the Atlantic Forest and Cerrado domains (states of Alagoas, Bahia, Maranhão, Paraíba, and Sergipe) and *Brejo de Altitude de Bonito* (Pernambuco state).

### ﻿ Hydroptila zerbinae

Taxon classificationAnimaliaTrichopteraHelicopsychidae

Souza, Santos & Takiya

5109DFFF-A9A2-513F-B786-1C4C1A78D656

Hydroptila zerbinae Souza, Santos & Takiya, 2014: 641 [type locality: Brazil, Pernambuco, Vicência Cachoeira do Engenho Embú, 07°37'22"S, 35°22'51"W, el. 186 m; DZRJ; male].

#### Material examined.

**Brazil: Pernambuco**: B1 (1 male); B2 (26 males); C1 (243 males); C3 (1 male); D7 (2 males); D8 (1 male).

#### Distribution.

Brazil (AL, BA, PE).

#### Remarks.

Previously recorded only in the Brazilian Northeast region, Caatinga and Atlantic Forest domains (states of Alagoas, Bahia, and Pernambuco) (Souza et al. 2014). This study expands its occurrence to the Brejos de Altitude.

## ﻿Discussion

The new species described here is an important step forward for the knowledge of Trichoptera in the Brejos de Altitude of Northeastern Brazil. Furthermore, the species presented here composes a small group of 12 of the 177 species of Helicopsyche (Feropsyche) that have all the semaphoronts described. Knowing and describing all semaphoronts represents a qualitative gain of information mainly for morphology-based systematics (to differentiate similar or cryptic species), and quantitative gain of characters for phylogenetic analyses, since different semaphoronts may represent distinct evolutionary scenarios (Farias et al. 2020).

This study is the first on the caddisfly biodiversity in the Brejos de Altitude, and a includes new species record from Brazil (*Cyrnelluskozepes*), and new records for the Brazilian Northeast region (*Cyrnelluskozepes* and *Metrichiapeluda*), and Pernambuco state (*Chimarrapotiguar*, *Cyrnelluskozepes*, *Cyrnellusfraternus*, and *Metrichiapeluda*), as well as a new species, *Helicopsycheralphi* sp. nov. Including the species previously recorded for Brazilian Northeast region (Santos et al. 2022) and Pernambuco state (França et al. 2013; Souza et al. 2013a, 2013b; Souza and Santos 2017; Gomes and Calor 2019; Pereira-Filho et al. 2020), 169 and 43 species have now been recorded from the Brazilian Northeast region and Pernambuco state, respectively. All species in this study, except *Chimarrapotiguar* and *Oxyethiratica*, constitute new records from the Brejos de Altitude. Among the species listed here, *S.palifera*, *Oecetisexcisa*, *Cyrnellusfraternus*, *Cyrnellusmammillatus*, and *Oxyethiratica* present disjunct distributions in the Atlantic Forest and Amazon rainforest. On the other hand, *Chimarrapotiguar*, *Metrichiapeluda*, and *Helicopsychezerbinae* have known distributions from the Atlantic Forest, and *Neotrichiafeolai* was known only from the Amazon rainforest (Santos et al. 2022). In this way, Brejo de Altitude de Triunfo seems to be a refuge for caddisflies with distributions in the Atlantic Forest and Amazon rainforest. The presence of Trichoptera with disjunct distributions in the Brejo de Altitude de Triunfo corresponds to a pattern registered for other taxa (e.g., Borges-Nojosa and Caramaschi 2003; Castro et al. 2019a; Silveira et al. 2019; Pereira-Filho et al. 2020).

Our results are helpful in guiding further studies in understanding the historical relationships between the Atlantic Forest and Amazon rainforest through the Brejos de Altitude. The shared distribution of these caddisfly species can be the result of past connections, when these enclaves acted as biological corridors between the Atlantic Forest and Amazon rainforest, harboring species from both domains, as proposed by some authors (e.g., Auler et al. 2004; Batalha-Filho et al. 2013; Silveira et al. 2019). In addition, our studies contribute to conservation strategies for the Brejos de Altitude. These areas are highly degraded due to deforestation, illegal hunting, and habitat fragmentation (Pereira-Filho et al. 2017). According to SOS Mata Atlântica & INPE (2019) and Pereira-Filho et al. (2020), these enclaves should be considered as the most threatened sector of the Atlantic Forest and conservation efforts are urgent.

Among the 43 Brejos de Altitude (Tabarelli and Santos 2004), except for this paper, there are only four caddisfly species recorded from these ‘’islands of humid tropical forests’’ in the Caatinga domain. Three of them from Brejo de Altitude de Bonito, Pernambuco state (*Macrostemumhyalinum*, *Oxyethiratica*, and *Atopsycheantisuya*) (França et al. 2013; Souza and Santos 2017; Gomes and Calor 2019), and one from Brejo de Altitude de Portalegre, Rio Grande do Norte state (*Chimarrapotiguar*) (Queiroz et al. 2020). Despite the increase in the number of species with this study, the number of collection sites remains insufficient, consequently taxonomic inventories and description of new species are important to fill gaps in taxonomic and biogeographic knowledge at the Brejos de Altitude.

## ﻿Conclusions

The present paper identified eleven caddisfly species from the Brejo de Altitude de Triunfo, Pernambuco state. These data revealed four new distributional records for Pernambuco state (*Chimarrapotiguar*, *Cyrnellusfraternus*, *Cyrnelluskozepes*, and *Metrichiapeluda*), two of them for Brazilian Northeast region (*Cyrnelluskozepes* and *Metrichiapeluda*), and one for Brazil (*Cyrnelluskozepes*). Previously, 39 species were registered for Pernambuco state and as a product of this survey, the records are updated to 43 species. Furthermore, this inventory is a pioneer in Brejos de Altitude, thus showing the lack of knowledge of the fauna of Trichoptera in these locations, which possibly have the dynamics of populations influenced by the isolation of these enclaves.

In addition, a new species of Helicopsyche (Feropsyche) is described, including all semaphoronts. In this way, this description represents a qualitative gain of information mainly for systematics based on morphology (Farias et al. 2020), as it presents a new source of characters for phylogenetic studies and also increases the accuracy in the identification of immatures and females, also useful in ecological studies.

## Supplementary Material

XML Treatment for Helicopsyche (Feropsyche) ralphi

XML Treatment for Smicridea (Smicridea) palifera

XML Treatment for Oecetis excisa

XML Treatment for Chimarra potiguar

XML Treatment for Cyrnellus fraternus

XML Treatment for Cyrnellus mammillatus

XML Treatment for Cyrnellus kozepes

XML Treatment for Metrichia peluda

XML Treatment for Neotrichia feolai

XML Treatment for Oxyethira tica

XML Treatment for Hydroptila zerbinae
